# Small Bowel Obstruction Caused by Type IV Hiatal Hernia

**DOI:** 10.1155/2024/8837649

**Published:** 2024-02-20

**Authors:** Katsudai Shirakabe, Naoya Ozawa, Yoshihiro Mochizuki, Ken Mizokami

**Affiliations:** Department of General Surgery, Tokyo Bay Urayasu Ichikawa Medical Center, 3-4-32 Todaijima, Urayasu City, Chiba, Japan

## Abstract

Type IV hiatal hernia of the esophagus is characterized by herniation of the stomach and associated organs, such as the spleen, large and small bowel, and pancreas, through the esophageal hiatus. It is a relatively rare form of hiatal hernia that sometimes requires emergency surgery due to gastric incarceration, volvulus, and strangulation. Of these, small bowel obstruction is extremely rare and requires surgery. We report the case of an 83-year-old woman who was admitted to the hospital for small bowel obstruction caused by an ileum that had incarcerated the esophageal hiatus; emergency laparoscopic surgery was performed.

## 1. Introduction

Hiatus hernias refer to the herniation of the elements of the abdominal cavity through the esophageal hiatus of the diaphragm. They are broadly divided into sliding and paraesophageal hernias. Among them, type IV hiatal hernia of the esophagus is characterized by the presence of organs other than the stomach (e.g., colon, spleen, pancreas, and small intestine) in the hernial sac, with a significant defect in the phrenoesophageal membrane.

Small esophageal hernias may be asymptomatic, while larger hernias may cause symptoms such as heartburn, belching, regurgitation, dysphagia, chest pain, and nausea due to acid reflux [1]. The estimated rate of hiatus hernias that develop acute symptoms requiring emergency surgery is 1.16% per year [2]. We report a very rare case of paraesophageal hernia in which the small intestine was incarcerated, and emergency surgery was performed.

## 2. Case Presentation

An 83-year-old woman presented to the emergency room complaining of frequent vomiting. According to the patient, she experienced a loss of appetite and heartburn that started a few days earlier, followed by vomiting that had begun earlier today. Furthermore, bowel movements had been absent for two days. The patients had comorbidities, such as moderate aortic valve stenosis, diabetes, and hypertension.

**Figure 1:**
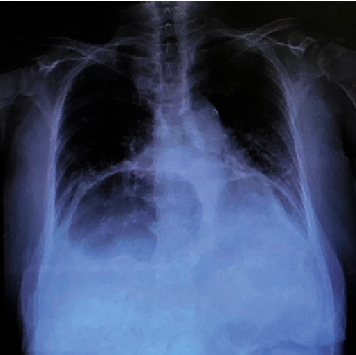
The mediastinum is enlarged, and gas-filled structures are observed.

**Figure 2:**
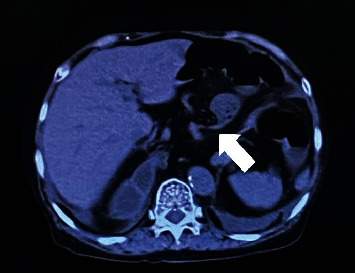
White arrow indicates caliber changes in the small intestine (axial section).

**Figure 3:**
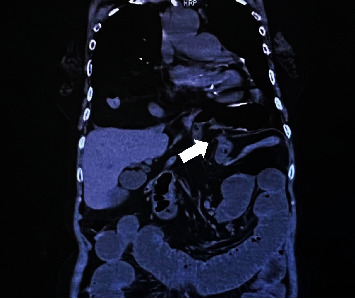
White arrow indicates changes in the caliber of the small intestine (coronal section).

The patient's vital signs were stable and afebrile. Physical examination revealed a distended abdomen but no tenderness or rebound tenderness. Laboratory findings showed acute kidney injury with a blood urea nitrogen value of 41.3 mg/dl and a creatinine value of 1.34 mg/dl. Chest X-ray and computed tomography revealed a type IV hiatal hernia of the esophagus (Figures 1 and 2), and the ileum was incarcerated, causing small bowel obstruction (Figures 2 and 3).

The patient was admitted to our hospital and underwent intensive fluid resuscitation and nasogastric tube decompression. On the second day of admission, the symptoms did not improve, and the patient underwent laparoscopic surgery.

**Figure 4:**
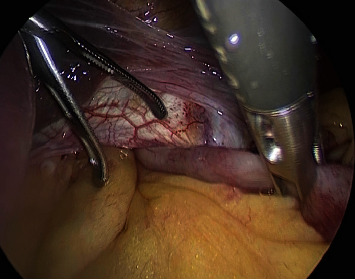
Incarcerated ileum in the esophageal hiatus.

**Figure 5:**
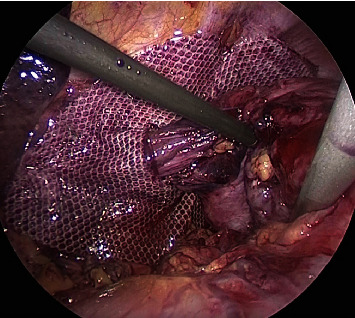
Esophageal hiatus being sutured and closed with mesh.

During the surgery, the incarcerated ileum was carefully reduced (Figure 4). There was no evidence of ischemia in the incarcerated ileum. Subsequently, an esophageal hiatal hernial repair was performed. The lower esophagus in the mediastinum was fully mobilized, suture closure of the esophageal hiatus was performed, and the mesh was fixed to the diaphragm (Figure 5). A Nissen fundoplication was then performed, and the operation was completed without gastric suture fixation.

The operative time was 4 hours 37 minutes. Postoperatively, the patient developed respiratory failure on the first postoperative day; she was intubated and required intensive care. The cause of respiratory failure was thought to be cardiogenic pulmonary edema due to excessive fluid infusion during surgery. The respiratory condition did not improve, and the patient developed complicated recurring pneumonia and died on the 32nd postoperative day.

## 3. Discussion

Hiatal hernia is a common condition characterized by protrusion of abdominal structures other than the esophagus into the thoracic cavity due to enlargement of the diaphragmatic hiatus.

In the early 1900s, attempts to classify hiatal hernias into subtypes began, and the current anatomical classification has evolved to divide hiatal hernias into types I to IV [3]. More than 95% of hiatal hernias are type I. Types II to IV are classified as paraesophageal hernias and are distinguished from type I hernias by the relatively preserved posterolateral attachment of the esophageal mucosa around the gastroesophageal junction [4].

Most paraesophageal hernias are symptomatic. However, in many patients, the symptoms are mild and often discovered incidentally on chest radiography performed for other purposes [5]. When carefully interviewed, patients often complain of chest fullness and shortness of breath after eating, while heartburn and reflux symptoms are rare in paraesophageal hernias.

Routine repair of asymptomatic paraesophageal hernias is not always recommended. Patient age and comorbidities should be considered when determining indications for surgery. However, all symptomatic paraesophageal hiatal hernias require repair. In particular, patients with symptoms of gastric incarceration or volvulus should undergo repair [6].

There is very little published information on the natural history of untreated paraesophageal hernias, but it has been suggested that the risk of progression from asymptomatic to symptomatic paraesophageal hernias is approximately 13.9% per year [2]. The recommended surgical procedures for paraesophageal hernia include laparoscopic hernia reduction, esophagogastric junction mobilization, repair of diaphragmatic defects, and intraperitoneal fixation of the stomach (sometimes with fundoplication or other antireflux procedures).

Since many patients are elderly and have medical comorbidities of varying severity, with a reported mortality rate of 1.38% for elective surgery [2], it is essential to consider the risks of surgery versus the risks of gastric volvulus and strangulation.

In addition, paraesophageal hernias can cause pulmonary edema and heart failure due to pulmonary venous obstruction. Preoperative echocardiography is important for evaluating cardiac function and assessing left atrial compression.

In some patients, definitive surgical repair is considered impossible. In such cases, endoscopic repair and percutaneous endoscopic gastrostomy are better options for avoiding open surgery [7]. However, it should be noted that this method has not been used to repair paraesophageal hernias.

The average mortality rate of emergency esophageal hernia surgery is approximately 17% [2]. Surgical mortality is primarily associated with pulmonary complications, thromboembolism, and bleeding [8]. The most common causes of emergency surgery are gastric entrapment, torsion, and strangulation, while small bowel obstruction due to incarceration of a hiatus hernia is a very rare acute complication of paraesophageal hernias. To the best of our knowledge, there are no other reports in the literature; therefore, further case accumulation and research are needed to provide optimal management and treatment for similar cases.

In this case, the patient had several medical comorbidities and a high operative risk. Although there are reports of gastric volvulus and strangulation that avoided emergency surgery by endoscopic reduction and fixation with percutaneous endoscopic gastrostomy, it was difficult to perform the endoscopic reduction in this case because the patient had a small bowel obstruction due to an internal hernia. In addition, various surgical approaches, techniques, and modalities for paraesophageal hernia repair may increase the anesthetic time and the risk of morbidity and mortality in elderly and frail patients.

Our patient was severely dehydrated due to small bowel obstruction, and the risk could have been reduced by performing only internal hernia reduction in emergency surgery and then performing esophageal hiatal hernia repair through elective surgery after the patient's general condition stabilized. In addition, laparotomy could have been chosen to reduce operative time if small bowel ischaemia was suspected.

In conclusion, emergency surgery for a hiatal hernia is associated with a high mortality rate. The most common causes are gastric incarceration, volvulus, or strangulation. Small bowel obstruction due to incarceration in the hiatus is extremely rare. When deciding on a treatment plan, it is important to consider the patient's comorbidities and surgical risks.

## Data Availability

All data regarding this case report has been reported in the manuscript. Please contact the corresponding author in case of requiring any further information.
